# Pomelo Peel Essential Oil Ameliorates Cerebral Ischemia-Reperfusion Injury through Regulating Redox Homeostasis in Rats and SH-SY5Y Cells

**DOI:** 10.1155/2022/8279851

**Published:** 2022-05-05

**Authors:** Wanxiang Hu, Menghua Chen, Wenyan Wang, Fan Huang, Xinyue Tian, Lu Xie

**Affiliations:** ^1^Department of Physiology, Pre-Clinical Science, Guangxi Medical University, Nanning, Guangxi 530021, China; ^2^Department of Intensive Care Unit, The Second Affiliated Hospital of Guangxi Medical University, Nanning, Guangxi 530000, China

## Abstract

**Background:**

In cardiac accident/cardiopulmonary resuscitation (CA/CPR) rat model, oxidative stress occurs during cerebral ischemia/reperfusion injury (CIRI), and antioxidative treatment has a neuroprotective effect. The antioxidant capabilities of pomelo peel essential oil (PPEO) have mostly been investigated in vitro, with little convincing data in vivo, particularly whether PPEO has a neuroprotective role against CIRI.

**Methods:**

In this investigation, a CA/CPR SD rat model and an oxygen-glucose deprivation/reperfusion (OGD/R) SH-SY5Y cell model were used to imitate the CIRI, and the neuroprotective role of PPEO was discovered in both. The morphological changes of neurons after PPEO treatment were observed using Nissl staining and transmission electron microscopy, while biochemical markers such as MDA, GSH, and Fe^2+^ were evaluated. Furthermore, western blot, immunofluorescence, and immunohistochemistry were used to examine the proteins GPX4, SLC7A11, ACSL4, and Nrf2.

**Results:**

Significant morphological alterations were identified during the pathological progression of CIRI. The neurologic deficit scores improved after PPEO therapy, and the expression of GPX4 and SLC7A11 increased, while the levels of intracellular Fe^2+^, ROS, and ACSL4 declined. PPEO also prevented CIRI caused by erastin (a specific inhibitor of SLC7A11) or RSL3 (inhibitor of GPX4). Furthermore, PPEO-induced increases in SLC7A11 and GPX4 may be related to Nrf2 translocation to the nucleus.

**Conclusions:**

In vitro and in vivo, we verified and investigated the neuroprotective effects of PPEO on CIRI. The underlying process may be connected to redox homeostasis regulation, which enhances antioxidative capacity through upmodulation of SLC7A11 and GPX4. It implies that PPEO will be considered as a source of potential adjuvant therapeutic agents for improving CIRI outcomes.

## 1. Introduction

Among the complicated physiopathologic mechanisms involved in cerebral ischemia reperfusion injury (CIRI), oxidative stress has always been regarded as a critical event which attributes to high mortality and permanent disability. The imbalance between oxidative stress and antioxidant capacity may directly induce many neurons death and irreversible neurological dysfunction [1]. Therefore, how to reduce neuron death and alleviate ischemia reperfusion injury should come first in the avenue of exploring the intricate mechanisms of CIRI. Also, focusing on modulating redox homeostasis in CIRI should promote discovering new pharmacological targets or exploring novel therapeutic agents.

In fact, growing evidence has suggested that supplements of exogenous antioxidants, especially those derived from plants or folk medicine, exert neuroprotective role through decreasing the oxidative stress in focal ischemia stroke [2, 3]. Our previous study found that green tea polyphenol, as a dietary antioxidant, decreased the mortality of CA/CPR rats and improved neurological deficit scores [4]. We also found that green tea polyphenol attenuates neuronal oxidative stress-related apoptosis through downregulating phosphorylation of the ERK and JNK signal pathways.

Similarly, as one of the citrus fruits favored by people worldwide, pomelo is widely grown and consumed in the South of China. Except for the edible part, most pomelo peels have been regarded as valueless and discarded. However, studies report that citrus and its products have good antioxidant properties because of its high content of active substances such as terpenes, aldehydes, esters, and ketones [5]. PPEO, a crude compound extracted from pomelo peel, is considered to have potential therapeutic effects for its antioxidant properties or other biological activities [6, 7]. In this study, we selected Shatian pomelo peculiar belonging to Rongxian, Guangxi, and extracted the essential oil from outer peel by distillation. GC/MS analysis showed that the main components of PPEO were terpenes and sesquiterpene, which exert potential antioxidant activities and are generally considered to be safe. Nevertheless, whether PPEO could provide neuroprotection against CIRI and whether the underlying mechanism maybe related to modulating redox homeostasis have not been explored. For this purpose, CA/CPR rat model and OGD/R cell model were used, respectively, to investigate the effects of PPEO on CIRI, even the underlying mechanism. PPEO, as a natural product from medicinal food, is expected to provide an important treatmental option for the treatment of acute global cerebral ischemia reperfusion injury.

## 2. Materials and Methods

### 2.1. Main Chemical and Reagents

The chemical and reagents used were Nissl stain solution (cresyl violet, G1430, Solarbio), malondialdehyde (MDA) assay kit; glutathione (GSH) assay kit, dimethyl sulfoxide (DMSO), BCA kits (Beyotime Biotechnology, Beijing), rabbit antibodies against antiglutathione peroxidase 4 (GPX4, ABclonal, USA, A13309), anticystine/glutamate antiporter (SLC7A11, ab175186, Abcom, USA), ACSL4 (sc-271800, Santa Cruz Biotechnology Inc., USA), Nrf2 (ABclonal, USA), GAPDH (1 : 1000, Cell Signaling Technologies, United States, #5174), tublin (1 : 1000, Cell Signaling Technologies, United States). Micro Malondialdehyde (MDA) Assay Kit (BC0025, Solarbio Science & Technology Co., Ltd., Beijing.), and Micro Reduced Glutathione (GSH) Assay Kit (BC1175, Solarbio Science & Technology Co., Ltd., Beijing). Iron Assay Kit was obtained from Nanjing Jiancheng Co. (A039-2, China). Reactive Oxygen Species Assay Kit was obtained from Beyotime Biotechnology (S0033, Beijing). Fetal bovine serum (FBS) was purchased from GIBCO Inc. (Logan, Utah, USA). Erastin (T1765, TargetMol, USA) and RSL3 (HY-100218A, MedChemExpress, USA) were also used.

### 2.2. Thermal Distillation of Essential Oil from Rongxian Pomelo Peel and GC/MS Analysis

Pomelo fruits (Citrus maxima (Burm.) Merr. cv. Shatian Yu) were selected from Rongxian in Guangxi province at harvest maturity, usually in November every year. After identification by Professor Yusong Huang from Herbarium (IBK), Guangxi Institute of Botany, Guangxi Zhuang Autonomous region, and Chinese Academy of Sciences, the cleaning epicarp of fresh fruit were cut into small pieces and soaked in distilled water for 4 h at least. The ratio of material/solvent is about 1 : 6. Through three times of thermal distillation, the upper oil phase (the crude essential oil of pomelo peel) was isolated by a Soxhlet extraction device and sealed and stored from light at 4°C for further use.

400 *μ*l sample solution was used to acquire the chemical composition by gas chromatography-mass spectrometry (GC/MS) analysis, which is performed using a GC/MS-Thermon 1300 (Thermo Corp., American) interfaced impact ionization (70 eV) and a TG-5 capillary columns (30 m × 0.25 mm × 0.3 *μ*m, Agilent Technologies, Folsom, CA, USA). The oven temperature was held at 50°C, for 3 min, and then increased at 6°C/min to 250°C for 5 min. The mass range were 40–650 amu. The injector, iron source, and band four temperature were set at 250°C, 230°C, and 200°C, respectively.

### 2.3. Experimental Animals and Cerebral Ischemia Reperfusion Modeling

A total of 90 adult male Sprague-Dawley (SD) rats aged 12-16 weeks and weighting 230-250 g were purchased from the Animal Center of Guangxi Medical University (Nanning, China). All of animal procedures was approved by the Animal Ethics Committee of Guangxi Medical University. After feeding with normal rat chow and water ad libitum and kept on a 12 : 12 h light-dark cycle in order to acclimatize for one week, all rats besides the sham group were induced into CA by esophageal electrical stimulation and then CPR and then randomly divided into the following five groups: the model group (NS group), the vehicle group (Gly group), and three PPEO-treated groups. The cardiac arrest/cerebral ischemia reperfusion model of SD rat was established according to the methods previously reported [8]. 0.9% saline, 10% glycerin solution, and different doses of pomelo essential oil (12.5, 25, and 50 mg/kg) were administered immediately after resuscitation via femoral vein, respectively. 24 hours after resuscitation, experimental rats were killed by intraperitoneal injection of excess sodium pentobarbital and brain tissues were harvested.

### 2.4. Neurological Evaluation In Vivo Experiments

Neurological dysfunction scores (NDS) were evaluated at 24 hours after ROSC according to the classical method established by Jia et al. [9, 10]. In brief, it contains level of arousal, cranial nerve reflexes, motor and sensory function, and behavior. The neurological function values were scored from a range of 0 to 80. The lower scores indicate more severe neurological deficits. Two independent investigators who blinded to this study measured the NDS in this evaluation.

### 2.5. Transmission Electron Microscopy

Rats were perfused rapidly with mixed perfusate (4% paraformaldehyde and 2.5% glutaraldehyde in 0.2 mol/l PBS). Several 1 mm^3^ samples were dissected from cerebral cortex layer near forehead and then fixed with 3% glutaraldehyde in phosphoric acid buffer for 4 h at least, followed by postfixation in 1% osmium tetroxide for 1 h, dehydration, and drying. The samples were sprayed with an IB5 (IB5) ion sputtering instrument to observe the ultrastructure changes. Images were captured under an transmission electron microscope (H-7650, Hitachi, Tokyo, Japan).

### 2.6. Slice Preparation for Nissl Staining

Rats were anesthetized with excess sodium pentobarbital by intraperitoneal injection and then transcardially perfused with 0.9% saline (NS) followed by 4% neutralized formalin for in situ perfusion fixation. Brain samples were collected and postfixed in 10% neutralized formalin overnight and then dehydrated routinely, embedded in paraffin, and sectioned into different thickness slices according to different detection needs.

Paraffin-embedded brain sections of 5 *μ*m were immersed in 0.05% cresyl violet stain solution at 56°C for about 1 hour after dewaxing. Being rinsed softly with distilled water, the sections were immersed in a Nissl differentiation solution until the desired color, then mountedwith a cover slip and examined under a light microscope. Five brain sections were randomly selected for each sample, and the data are expressed as the number of Nissl bodies/field.

### 2.7. Immunohistochemistry and Immunofluorescence Detection

For immunohistochemical staining, 3 *μ*m slides were deparaffinized and rehydrated in PBS followed by blocking the endogenous peroxidase with 3% hydrogen peroxide. To avoid nonspecific reaction with primary antibody, slides were pretreated with 15% normal goat serum before sequentially incubated with the primary antibodies (GPX4, 1 : 300; SLC7A11, 1 : 100) and biotinylated secondary antibody. At last, signal amplification and detection were carried out by DAB coloring solution according to manufacturer's instructions and observed under an optical microscope. We collected data from at least five rats in each group. The area of positive cells of GPX4 or SLC7A11 was detected by Image-Pro Plus 6.0 software.

To clarify the relationship between GPX4 and ACSL4, we also applied double immunofluorescence staining to detect the expression level of these two proteins. 3 *μ*m slides were dewaxed, repaired, sealed, and then incubated with the primary antibodies for GPX4 (1 : 500) and ACSL4 (1 : 200) at 4°C. In the next day, the secondary antibodies (Alexa Flour 488 and Alexa Flour 555, 1 : 1000 dilution) were added and incubated for 1 h at 37°C, followed by washing with PBST three times. Then, the sections were protected by cover slips with antifading mounting medium containing DAPI. Normal rabbit/mouse IgG served as the negative control for the immunofluorescence assay. Also, immunofluorescence detection was used to assay the possibility that Nrf2 translocated to nucleus after OGD/R or PPEO treatment in SH-SY5Y cells. Finally, sections were observed by a fluorescence microscope, and the relative fluorescence intensity was analyzed by Image-Pro 6.0 software (Media Cybernetics, Silver Spring, MD).

### 2.8. Determination of Malondialdehyde, Iron, and GSH Content

A certain amounts of fresh brain tissue of different groups were taken at 24 h after ROSC, then homogenized in ice-cold PBS, and centrifuged at 8000 × *g* for 10 min at 4°C. The supernatants were used to measure the levels of malondialdehyde (MDA) and intracellular glutathione (GSH). The measurement steps performed refer, respectively, to manufacturer's instructions of Micro MDA Assay Kit and Micro Reduced GSH Assay Kit. MDA concentrations were measured at 450 nm, 532 nm, and 600 nm by microplate reader (Multiscan MK3, Thermo, USA) and expressed as nanomoles per milligram protein, while GSH level was detected at 412 nm and calculated in reference to a glutathione standard curve.

Iron content in fresh brain tissue was determined by Iron Assay Kit (A039-2, Nanjing Jiancheng, China) according to the respective manufacturer's instructions. In brief, every 100 mg fresh rat brain cortex was homogenized in 900 *μ*l saline, the supernatant was obtained after being centrifuged at 3500 × *g* for 10 min for the iron assay. As the same aim, collected cells were added iron assay buffer following ultrasonication, and then, the supernatant was collected after centrifugation (16000 × *g*, 10 min, 4°C) for the iron analysis. Half supernatant was added reduce lysates to reduce ferric iron (Fe^3+^) to ferrous iron (Fe^2+^) and incubated for 30 min at room temperature in the dark. The iron reaction produced a colored complex and absorbance was measured at 593 nm. Intracellular iron concentration was expressed as micromoles per gram protein and measured as the ratio between Fe^2+^ and total iron (Fe^2+^ and Fe^3+^) according to the standard curve.

### 2.9. SH-SY5Y Cell Culture, Establishment of OGD/R Model, and Treatment

To simulate CIRI in vitro, an oxygen and glucose deprivation reperfusion model was used in SH-SY5Y cells. SH-SY5Y cells were cultured in Dulbecco's modified Eagle's medium supplemented with 10% fetal bovine serum (FBS) at 37°C under a humidified atmosphere of 5% CO_2_ and 95% air in this study. On reaching 70%~80% confluence, the culture medium was replaced with DMEM (without glucose) within a anaerobic container at 37°C to initate OGD insult except control group. Two hours later, the medium replaced again with NS, glycerin, and different concentrations of PPEO (2.5 mM, 5 mM, and 10 mM) according to the different groups. The cells in the positive control group were treated with erastin (10 *μ*M). All of the agents were incubated with the cells for 12 h.

### 2.10. Flow Cytometric Measurement of Intracellular Reactive Oxygen Species (ROS) Content

Intracellular ROS was measured by Reactive Oxygen Species Assay Kit (S0033, Beyotime Biotechnology, Beijing) with flow cytometer (Becton Dickinson FACS Calibur Cytometer, BD Biosciences, USA) following the provided procedure. Briefly, the cells were washed with PBS after collected and then resuspended in DCFH-DA solution at a dilution ratio of 1 : 1000 and incubated for 20 min at room temperature. Each sample was washed for three times and the cells were resuspended in 300 *μ*l serum-free MEM. The fluorescence intensity was recorded at 488 nm excitation wavelength and 525 nm emission wavelength by flow cytometry. The median fluorescence intensity (MFI) from 5 random fields was measured using Image-Pro Plus 6.0 software, and the MFI was used as an indicator to determine the amount of ROS.

### 2.11. Protein Extraction and Western Blot Analysis

Samples from the cells and animals were lysed in RIPA lysis buffer containing protease inhibitor and phosphatase inhibitor. The proteins were quantified by BCA kits (Beyotime Biotechnology, Beijing). Proteins were separated on a 12% gel and 10% gel transferred onto a polyvinylidene fluoride (PVDF) membrane (Bio-Rad, Hercules, CA, United States). The membranes were blocked for 40 min with 5% (*w*/*v*) milk dissolved in 0.1% Tween-20 in TBS at room temperature and then were incubated overnight at 4°C with the following primary antibodies: GPX4 (1 : 1000), SCL7A11 (1 : 1000), tublin (1 : 1000, ABclonal, United States, A12289), and GAPDH (1 : 1000). Subsequently, the membranes were washed thrice with TBST, and the membranes were incubated with horseradish peroxidase-conjugated secondary antibodies rabbit/mouse polyclonal antibody for 60 min. Odyssey two-color infrared fluorescence imaging system (LI-COR, United States) was used to visualize the signals. Image-Pro Plus software was used to analyze the relative band densities, and the band densities of target proteins were normalized to that of GAPDH or tublin. All experiments were repeated at least in triplicate.

### 2.12. Statistical Analysis

The obtained data were analyzed using GraphPad Prism 6 software. The results were presented as means ± standard deviation (SD). Significant differences between groups were evaluated by one-way analysis of variance (ANOVA). Data analysis was performed by software SPSS 22.0 (IBM Corporation, Armonk, NY, USA), and *P* value < 0.05 was regarded as statistical significance.

## 3. Results

### 3.1. Composition Analysis of PPEO by GC-MS

**Figure 1:**
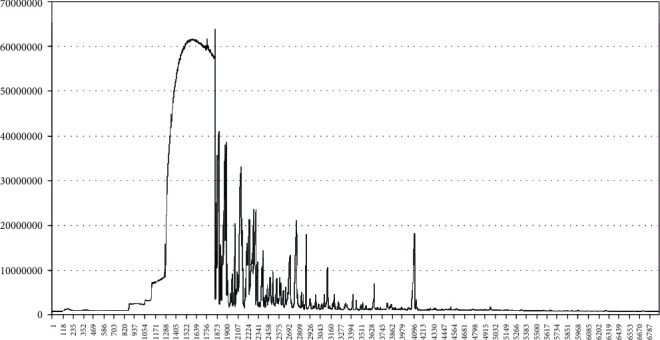
Gas chromatogram spectrum of PPEO from Rongxian in Guangxi province.

The PPEO obtained was pale yellow in color and a transparent liquid with a pleasant scent. The total ion chromatogram of PPEO obtained from GC/MS analysis is shown in Figure 1. There were 37 compounds identified. According to GC-MS analysis and NIST mass spectral data, the major constituents were terpene compounds, such as D-limonene (50.67% of all), *α*-pinene (4.01%), and *β*-pinene (1.96%)), as well as oxygenated derivatives (such as limonene oxide, cis-), and a tiny amount of alcohol, esters, and aldehydes. Only 0.97 percent of the sesquiterpene hydrocarbons were 2,6-octadien-1-ol, 3,7-dimethyl- (see Table 1 for details). The findings were comparable to those of previous studies. Differences in limonene content could be attributable to provenance, growth environment, and isolation methods.

### 3.2. PPEO Alleviated Neurological Impairment in CA/CPR Rats

The neurological dysfunction was partly due to the neuron death or dysfunction after CIRI.

**Figure 2:**
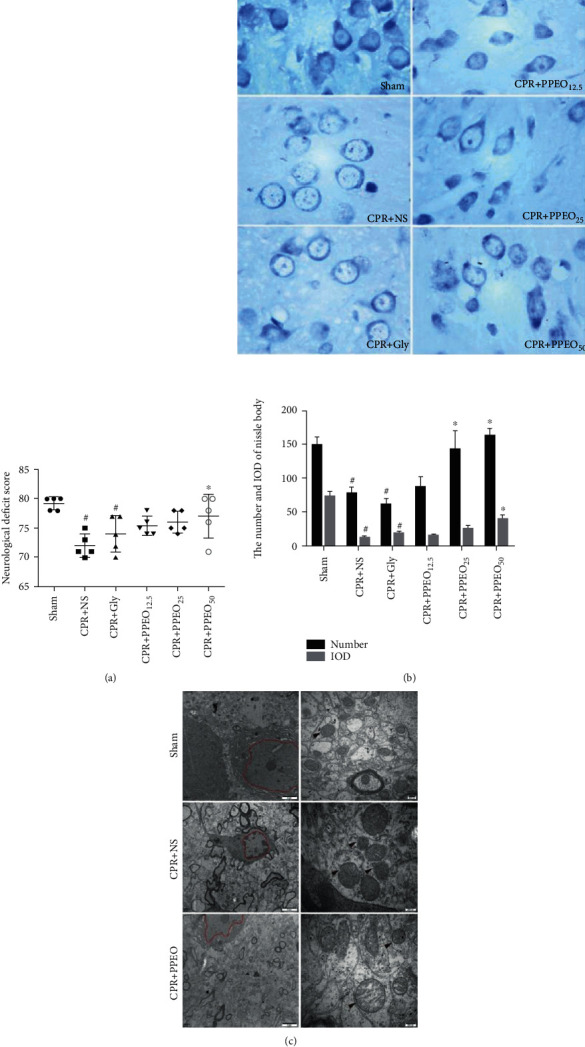
(a) Neurological function scores at 24 h after CA/CPR. All datas were represented as mean ± standard deviation. (b) Morphological changes of neurons by Nissl staining, the bar graph of the number and IOD of Nissl bodies, magnification ×40; ^#^compared to the sham group *P* < 0.05 and ^∗^compared to the CPR+NS group *P* < 0.05. (c) Morphological changes of mitochondria and nucleus observed by transmission electron microscopy (TEM). Black arrows showed the membrane of mitochondria. Compared to the sham group, the structure of mitochondria membrane was disorganized even ruptured in the model group. Scale bar: 2 *μ*m (left) or 200 nm (right).

Besides neurological function scores, we also observed the neuron damage through Nissl staining which assesses the dysfunction of protein synthesis in neurons. As shown, Figure 2(a) is neurological function scores at 24 h after CA/CPR; the CPR+NS group and CPR+Gly group showed the lower scores compared to the sham group, but the scores were improved in the PPEO-treated groups, especially in the CPR+PPEO_50_ group. It means the neurological function restored in some extent after administration of PPEO. In Figure 2(b), compared with the sham group, a significant loss of neurons, palely stained nuclei, and swollen and vacuole-like change were observed in the NS group and Gly group. The number and IOD value of Nissl bodies all increased after treatment of pomelo peel essential oil (*P* < 0.05) and showed the deeper stained as a whole.

Ultrastructural changes of neurons in different groups were examined by TEM. In contrast to the sham group, the CA/CPR model group exhibited electron lucent cytoplasm and more mitochondrial injury with swelled, large vacuoles, damaged cristae, and blurred membrane of mitochondria at the ultrastructural level. The ischemia reperfusion injury exerts nuclear membrane disappearance and nucleoplasm was uneven; some severely damaged neurons appeared fragmented nucleolus. As expected, we also observed that structures of neurons and mitochondria both are improved after treatment of PPEO at dose of 25 mg/kg, as shown in Figure 2(c). These results suggested that PPEO alleviated the neuronal and mitochondrial damage induced by CIRI from morphological observation.

### 3.3. The Effect of PPEO on Modulating Redox Balance through Increasing the Expression of SLC7A11 and GPX4

**Figure 3:**
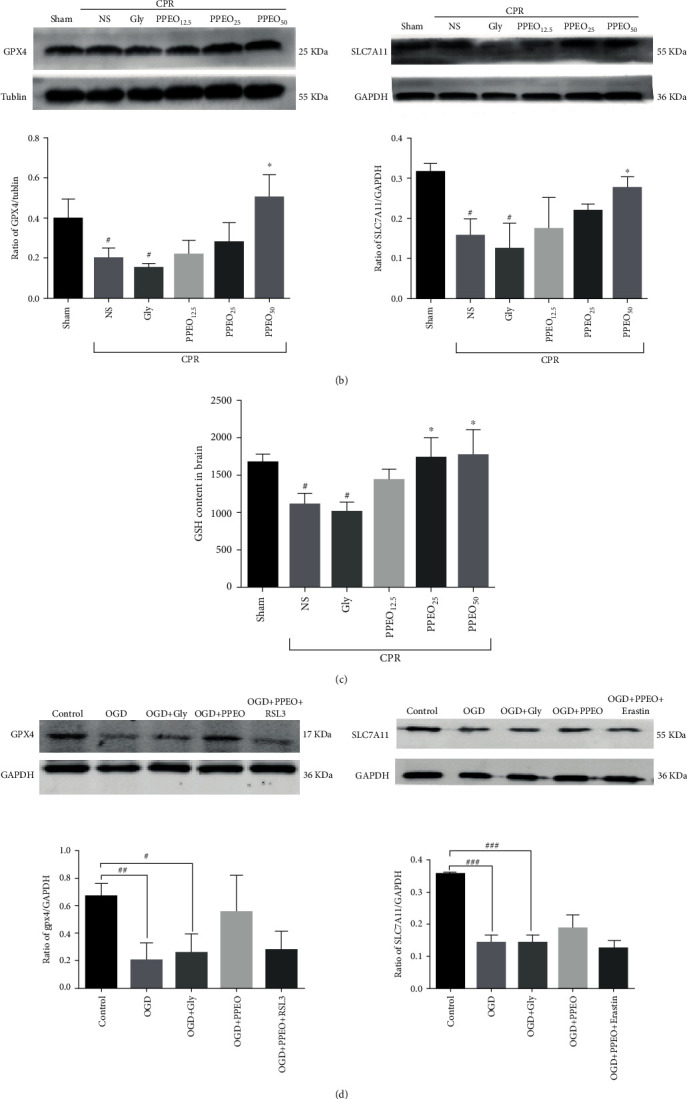
(a) Immunohistochemistry staining for SLC7A11 and GPX4 in cortex neurons (400x) and statistical analysis bar graph in each group, respectively. SLC7A11 or GPX4-positive neuron was stained brown. The data were expressed as the mean ± SEM (*n* = 5 per group). ^#^*P* < 0.05 and ^##^*P* < 0.01 compared to the sham group; ^∗^*P* < 0.05 and ^∗∗^*P* < 0.01 compared to the NS group. (b and d) Western blot analysis of GPX4 and SLC7A11 protein expression in vivo and in vitro. The data were expressed as the mean ± SEM (*n* = 5 per group). ^#^*P* < 0.05 compared to the sham group; ^∗^*P* < 0.05 compared to the NS group. (c) Measurement of the level of GSH in different groups or SH-SY5Y cells. ^#^*P* < 0.05 compared to the sham group and ^∗^*P* < 0.05 or ^∗∗^*P*<0.01 compared to the NS group. (e) GSH content assay in SH-SY5Y cells. ^#^*P* < 0.05 or ^##^*P* < 0.01 compared to the control group and ^∗∗^*P*<0.01 compared to the OGD+PPEO group.

Redox imbalance is inevitably involved in pathophysiological process of CIRI after CA/CPR. We detected oxidative stress-related proteins such as GPX4 and SLC7A11 by western blotting analysis and immunohistochemistry staining. As illustrated in Figure 3(a), there were not any histopathological abnormalities in the sham group; however, the area of GPX4 and SLC7A11 immunopositive cells in the NS group is decreased but it elevated markedly after PPEO treatment (*P* < 0.05). Western blot results also indicated that PPEO inhibits GPX4 and SLC7A11 expression after CIRI, especially in the PPEO_50_ group (Figure 3(b), *P* < 0.05). Furthermore, the neuroprotective role of PPEO was confirmed in vitro (Figure 3(d)). Especially, the decrease of GPX4 and SLC7A11 levels caused by injury can be neutralized by RSL3 or erastin, respectively. Also, the levels of GSH both in brain tissue and SH-SY5Y cells are decreased obviously after CIRI or OGD/R but increased after PPEO treatment (see Figures 3(c) and 3(e)). These findings suggested that the neuroprotective role of PPEO maybe through rebuilding balance of redox via modulation of SLC7A11 and GPX4.

### 3.4. PPEO Improved Lipid Peroxidation Induced by CA/CPR or OGD/R

**Figure 4:**
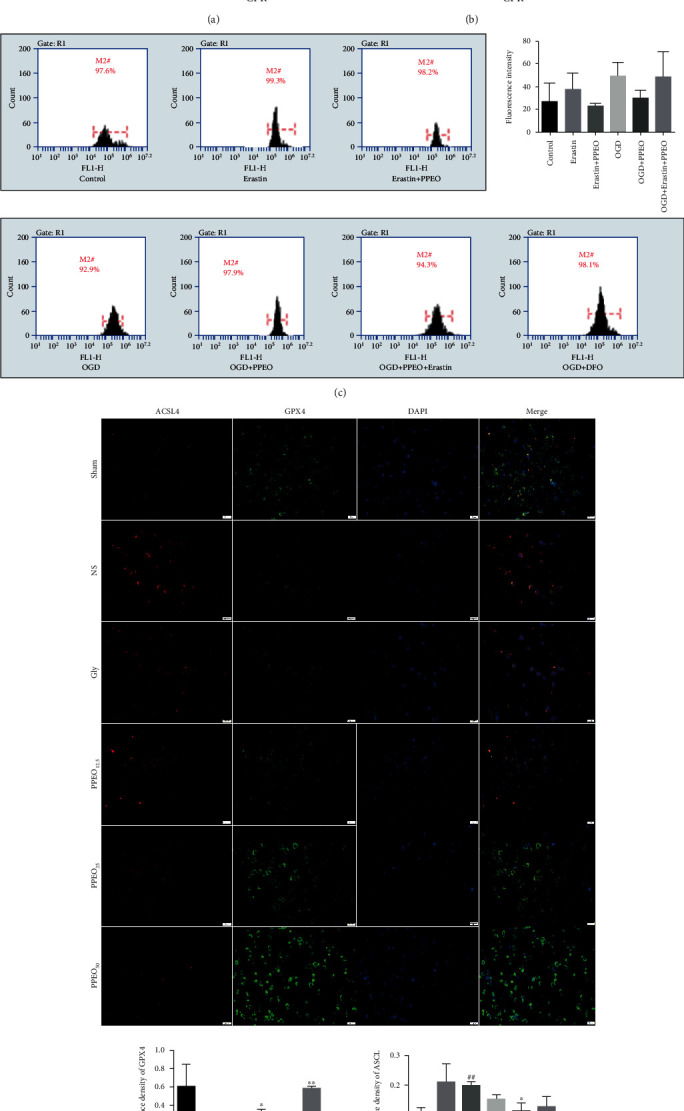
(a and b) Measurement of the level of MDA and total iron in different groups. (c) Flow cytometry assay of intracellular ROS in SH-SY5Y cells. (d) Immunofluorescence double staining of ACSL4 and Gpx4 (scale bar: 20 *μ*m). ^#^*P* < 0.05 or ^##^*P* < 0.01 compared to the sham group and ^∗^*P* < 0.05 or ^∗∗^*P* < 0.01 compared to the NS group. The data were expressed as the mean ± SEM.

The ameliorative effects of PPEO on lipid peroxidation were also investigated on activities of GSH and iron contents in fresh brain samples by corresponding kit assay. As shown in Figure 4(a), the contents of MDA, the end products of lipid peroxidation, were increased in the model group and Gly group (*P* < 0.05) but significantly decreased following the treatment with pomelo peel essential oil at dose of 50 mg/kg (*P* < 0.05). Similar results were observed in iron content detection (Figure 4(b)).

Based on datas described above, we further confirm the inhibitory effect of PPEO on lipid peroxidation in vitro. ROS level was chosen to evaluate neuronal injury and directly reflect the degree of oxidative stress in cells. In current study, we discovered a significant increase of ROS level in SH-SY5Y cells after OGD/R injury, and the ROS production was slightly attenuated after treatment with PPEO. Moreover, erastin which inhibited SLC7A11 mainly abrogated the effect of PPEO (Figure 4(c)).

We also detected ACSL4 and GPX_4_ by immunofluorescence staining. As shown in Figure 4(d), the GPX_4_ expression significantly decreased after CA/CPR that was consistent with previous study, while ACSL4 increased compared to the sham group (*P* < 0.05). PPEO protected the CA/CPR rats against CIRI through decreased the expression of ACSL4 and simultaneously rescuing the expression of Gpx4, especially in the dose of 50 mg/kg (*P* < 0.05). The above findings suggested again that PPEO alleviated CIRI through affecting lipid peroxidation.

### 3.5. PPEO Triggered the Expression of Nrf2 and Promoted Nrf2 Transporting into the Nucleus

**Figure 5:**
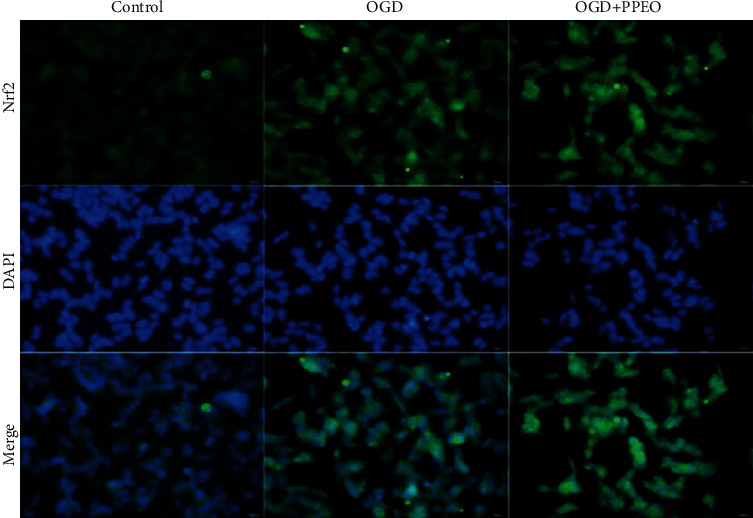
Immunofluorescence images of Nrf2-positive protein in nucleus after OGD/R or PPEO treatment. Scale bar: 50 *μ*m.

To elucidate why did SLC7A11 and GPX4 expression increase after PPEO treatment, we explored the upstream factors-Nrf2. The level of Nrf2 into the nucleus is shown in Figure 5 by the overlapping green and blue fluorescence. The more overlapping region there is, the more Nrf2 gets into the nucleus. We observed that Nrf2 level in cytoplasmic was increased after OGD/R compare to the control group, and fluorescence intensity of Nrf2 in nucleus significantly enhanced after PPEO treatment vs. OGD group. We have only observed this phenomenon, and more reliable data needs to be acquired in further experiments.

## 4. Discussion

Oxidative stress is an essential mechanism concerned with the pathophysiological process of cerebral ischemia reperfusion injury. Accumulating literatures report that some agents with antioxidant properties have a neuroprotective role against ischemic stroke [11–13]. Similarly, our previous study indicated that antioxidative therapy exerted an effective role against CIRI in CA/CPR rat model [14]. Some studies on components of citric fruits support the antioxidant, anti-inflammatory, and antinecroptosis effects in several experimental models. We focus on the antioxidative effect of PPEO on CIRI in the present study. Firstly, we extracted PPEO from Rongxian in Guangxi province and identified 37 kinds of components. Among them, limonene, the most abundant component which accounts for 50.67%, is easy to be oxidized to limoned-1,2-oxidation, carvone, or carvacrol which is reported that markedly inhibited oxidative stress-induced damage on hippocampal neurons death [15]. We hypothesized that limonene with antioxidant properties may be the functional component. The capacity of PPEO to act as antioxidants against CIRI was then demonstrated in vivo and in vitro through upmodulating SLC7A11 and GPX4 levels. Furthermore, the increased antioxidant capacity partly is related to promotion of Nrf2 translocation.

As a critical event occurs in CIRI, oxidative stress causes the generation of a lot of free radicals, which leads to membrane lipid peroxidation and neurons are damaged or even die. In many CIRI animal experiments, neurological dysfunction score (NDS) is used as a simple method for potential prognostic value and physiologic basis. We used NDS to identify the protective effect of PPEO and determine the efficacy and safety dose at 50 mg/kg. In fact, different dose of PPEO was adopted due to different research aim in our other studies in the same period [6, 7]. Nissl staining and electron microscopy were used to observe the protective effect of PPEO from general to ultrastructural level, respectively. Morphological results showed that PPEO really plays a protective role by promoting the recovery of neuronal Nissl bodies, increasing the function of neuronal protein synthesis, and upregulating some proteins expression against CIRI.

Then, focusing on the SystemXc^−^-GSH-GPX4 axis, SLC7A11 and GPX4 levels were investigated in vivo and in vitro. SLC7A11 is a core component of SystemXc^−^ which is in charge of the reversal transportation of intracellular glutamate and extracellular cystine [16]. Under physiological conditions, intracellular cystine is reduced to cysteine, which is involved in the synthesis of intracellular GSH. So, inhibition of SLC7A11 will cause decreasing of intracellular GSH level [17]. As an antioxidant defense enzyme, GPX4 can reduce toxic phospholipid peroxides (PL-OOH) to nontoxic lipid alcohols via using GSH as the substrate or electron donor [18]. Immunohistochemistry and western blot results in vivo demonstrated that the decrease of GPX4 and SLC7A11 induced by CIRI can be partially reversed by PPEO, and the similar effect was detected in vitro; furthermore, inhibitor of SLC7A11 and GPX4 seems to have the tendency to counteract the effect of PPEO, respectively. More interesting, the enhanced expression of SLC7A11 and GPX4 caused by PPEO can be reversed again by erastin (a small molecule compound with specific inhibitory effect on SLC7A11) and RSL3 (inhibitor of GPX4), respectively. Simultaneously, we also detected the GSH level of brain tissue homogenization and cells; both results indicate that PPEO increases the GSH contents after CIRI. And in vitro, we found again that erastin counteracts the increasing effect of PPEO on GSH after OGD/R treatment. All of the results above indicated that the neuroprotective mechanism of PPEO is related to enhancing the antioxidant capacity of cells by increasing the expression of SLC7A11 and GPX4 and enhancing the content of GSH, thus being conducive to maintaining the redox balance of neurons.

Brain tissue is a special organ vulnerable to oxidative stress due to some characteristics such as more contents of unsaturated fatty acids, high consumption of oxygen, and also rich blood flow. In addition, abundant nonheme irons in the brain accelerate membrane lipid peroxidation by Fenton reaction [19]. Some chemical and molecular biological indexes of lipid peroxidation were investigated for evaluating oxidative stress-induced damage. MDA, a final metabolite product of lipid peroxidation, is used to evaluate the severity of oxidative stress due to its stable chemical structure [20]. Our results in vivo showed that 50 mg/kg PPEO attenuates MDA contents of homogenate of brain tissue induced by CIRI. Secondly, we explored the inhibition effect of PPEO on intracellular ROS in SH-SY5Y cells after OGD 2 h. ROS is a kind of small molecule with high reactivity, including superoxide anion and hydrogen peroxide, hydrogen, and oxygen anion. ROS can receive an electronic to form stable polymers. Under physiological state, generation or inactivation of endogenous ROS is usually modulated by the endogenous antioxidants such as superoxide dismutase (SOD), GSH, and peroxidase (GPXS). When CIRI occurs, ROS increases explosively through multiple ways such as Fenton reaction induced by iron overload and mitochondrial source. When the generation of ROS is far beyond the scavenging capacity of the cell, it eventually leads to excessive accumulation of ROS. Especially, ·OH and ONOO- are prone to action with PUFA on the membrane which leads to lipid peroxidation reactions [21, 22]. Flow cytometry data in this study indicated that PPEO appears to decrease ROS increased by erastin treatment as well as OGD/R treatment and that erastin offsets PPEO effects, implying that PPEO modulates ROS formation during CIRI possibly through a crucial factor-SLC7A11. But regretfully, more data is required to reach statistical significance. It also reminds us that a variety of sources cause an increase in ROS as a result of OGD/R treatment. To explore the confusion, lipid ROS detection may become the focus in further study. Finally, we found that iron contents in brain tissue homogenate increased after CIRI, which is consistent with a recent study that demonstrated iron-dependent lipid peroxidation in CIRI [15]. However, there are no evident dose-dependent effects of PPEO on iron levels, which may be due to the multiple constituents contained in PPEO. So, it is worth exploring to find out which component in PPEO plays a key function in iron metabolism homeostasis.

**Figure 6:**
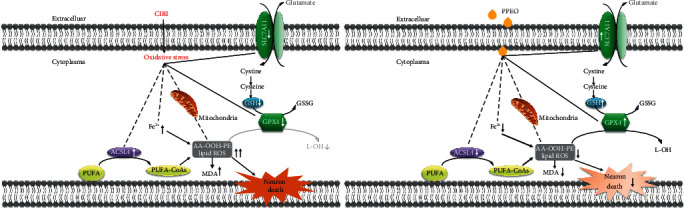
Many targets involved in the modulating mechanism of PPEO on ferroptosis in CIRI.

As previously stated, lipid metabolism homeostasis is determined by how to maintain the balance between generation and elimination of lipid peroxides. We are also concerned about the upstream source of lipid peroxidation, in addition to the downstream problem of lipid metabolism. It means that limiting the formation of lipid peroxides can protect neurons from lipid peroxidation damage. The enzyme acyl-CoA synthetase long-chain family member 4 (ACSL4) esterifies free polyunsaturated fatty acids (PUFAs) into PUFA-CoA, which are susceptible to oxidizing and being the main substrate for lipid peroxidation [23]. By using double immunofluorescence staining, we found that PPEO decreased ACSL4 expression while boosting GPX4 expression. The above findings suggest that, at some appropriate dose, PPEO has a broad effect on a variety of targets in redox-related pathway (see Figure 6 for details). For the first time, we used in vivo and in vitro ischemia-reperfusion models to highlight the neuroprotective effects of PPEO by boosting endogenous antioxidant function and lowering lipid peroxidation.

**Figure 7:**
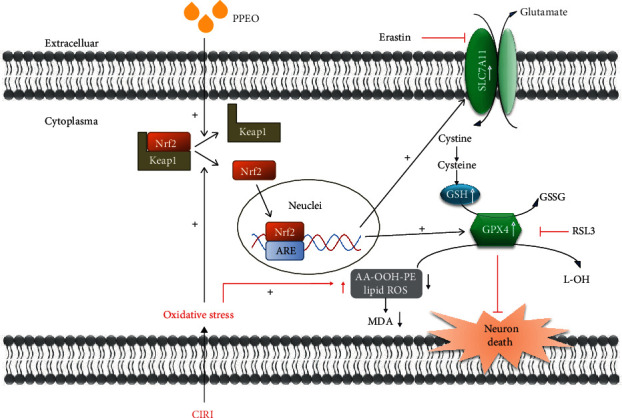
Mechanism of PPEO against CIRI through Nrf2-SystemXc^−^-GSH-GPX4 axis.

In addition, what caused the increase of GPX4 and SLC7A11 after PPEO treatment? The nuclear factor erythroid 2-related factor 2 (Nrf2) was chosen as the preferred candidate after a review of literatures revealed that Nrf2 is critical for maintaining intracellular oxidation homeostasis [24]. Oxidative stress can promote Nrf2 to be released from the Keap1-Nrf2 complex in the cytoplasm and translocated to the nucleus, where it then binds to the antioxidant response element (ARE) and promotes antioxidant gene transcription [25]. Marcos Roberto de Oliveira demonstrated that, in SH-SY5Y cells, naringenin (NGN) protected mitochondrial in a Nrf2/GSH-dependent way against mitochondrial toxicant [26]. Using the immunofluorescence method, we observed that the level of nucleus Nrf2 in the model group was significantly higher than that in the control group. Although a small number of Nrf2 translocate from the cytoplasm to the nucleus can be considered as an important response to oxidative stress, it is not enough to counteract the damage to protein synthesis, so we found that SLC7A11 protein expression is decreased in the OGD/R group. Furthermore, after treatment with PPEO, the fluorescence intensity of Nrf2 in the nucleus was significantly higher than that in the OGD/R group. It is indicated that the more translocation of Nrf2 trigged by PPEO exert positive effect, promoting more SLC7A11 expression and it is enough to boost intracellular antioxidative activity favoring to maintain the redox homeostasis, thereupon attenuated the damage to OGD/R cells (Figure 7). Surely, further studies should be performed to support this opinion.

Finally, there are several limitations in the current study. As a complex blend of numerous components, PPEO was investigated as whole in present study and our focus is only about its neuroprotective effects. However, it is unknown whether this is also the case when compared to one or two exact functional monomer such as limonene or pinene, or whether these monomer constituents interacted with each other. Secondly, our results suggested that the neuroprotective effects of PPEO maybe related to the upregulated Nrf pathway, but without substantial evidence supporting that. Further works are required to verify that by various means such as gene knockdown or overexpression, using an inhibitor as a control. In addition, iron overload may accelerate the production of ROS in mitochondria through Fenton reaction, which brought more serious injury after reperfusion, so intracellular iron needs to be measured in further experiments but not only tissue iron contents. At last, our research provided an experimental basis in utilization of the peel of Citrus which is usually discarded as a useless part of agricultural cash crops. It is beneficial to exploit natural health source products on preventing or treating cerebral ischemia injury.

## 5. Conclusion

To summarize, we present the first evidence that PPEO has a neuroprotective effect against CIRI in vivo and in vitro. We discovered that PPEO boosts endogenous antioxidative capacity by promoting translocation of Nrf2 from cytoplasm to nucleus which increases SLC7A11 and GPX4 expression. As a result, by modulating the redox balance, PPEO can prevent acute global cerebral ischemia-reperfusion injury caused by CA/CPR.

## Figures and Tables

**Table 1:** Volatile constituents of essential oil of pomelo peel from Rongxian in Guangxi province.

No.	Name of compound	Mol weight (amu)	Molecular formula	Relative content (%)
1	1-Butanol, 2-ethyl-	102.104	C_6_H_14_O	0.37
2	*β*-Pinene	136.125	C_10_H_16_	0.61
3	D-Limonene	136.125	C_10_H_16_	50.67
4	2-Furanmethanol, 5-ethenyltetrahydro-.alpha.,.alpha.,5-trimethyl-, cis-	170.131	C_10_H_18_O_2_	0.50
5	Benzene, 1-methyl-4-(1-methylethenyl)-	132.094	C_10_H_12_N_2_O_2_	1.71
6	1,6-Octadien-3-ol, 3,7-dimethyl-	154.136	C_10_H_18_O	5.03
7	Cyclohexane, 2-ethenyl-1,1-dimethyl-3-methylene-	150.141	C_10_H_18_	0.63
8	2-Cyclohexen-1-ol, 1-methyl-4-(1-methylethenyl)-, trans-	152.12	C_10_H_16_O	0.40
9	Limonene oxide, cis-	152.12	C_10_H_16_O	4.51
10	Cyclohexanol, 1-methyl-4-(1-methylethenyl)-	154.136	C_10_H_18_O	0.54
11	Bicyclo[3.3.0]oct-2-en-7-one, 6-methyl-	136.089	C_8_H_9_ClO	0.37
12	3-Cyclohexen-1-ol, 4-methyl-1-(1-methylethyl)-	154.136	C_10_H_18_O	1.56
13	Ethanone, 1-(4-methylphenyl)-	134.073	C_9_H_10_O	0.35
14	3,9-Epoxy-p-mentha-1,8 (10)-diene	150.104	C_10_H_14_O	0.65
15	1R-*α*-Pinene	136.125	C_10_H_16_	4.01
16	Santolina triene	136.125	C_10_H16	2.58
17	2-Cyclohexen-1-ol, 2-methyl-5-(1-methylethenyl)-, cis-	152.12	C_10_H_16_O	2.34
18	2,6-Octadienal, 3,7-dimethyl-, (Z)-	152.12	C_10_H_16_O	1.67
19	2-Cyclohexen-1-ol, 2-methyl-5-(1-methylethenyl)-, trans-	152.12	C_10_H_16_O	1.88
20	2-Cyclohexen-1-one, 2-methyl-5-(1-methylethenyl)-, (S)-	150.104	C_10_H_16_O	2.88
21	2,6-Octadien-1-ol, 3,7-dimethyl-	154.136	C_10_H_18_O	0.97
22	2,6-Octadienal, 3,7-dimethyl-	152.12	C_10_H_16_O	0.74
23	2-Cyclohexen-1-one, 3-methyl-6-(1-methylethenyl)-	150.104	C_10_H_14_O	0.81
24	1-Cyclohexene-1-methanol, 4-(1-methylethenyl)-	152.12	C_10_H_16_O	0.67
25	4-(1-Hydroxyethyl)benzaldehyde	150.068	C_9_H_10_O_2_	0.64
26	1,3,3-Trimethyl-2-hydroxymethyl-3,3-dimethyl-4-(3-methylbut-2-enyl)-cyclohexene	222.198		0.43
27	1,2-Cyclohexanediol, 1-methyl-4-(1-methylethenyl)-	170.131	C_10_H_18_O_2_	0.34
28	Cyclopropanecarboxaldehyde, 2-methyl-2-(4-methyl-3-pentenyl)-, trans-(.+-.)-	166.136	C_11_H_20_O	0.34
29	Bicyclo[3.1.1]heptane, 6,6-dimethyl-2-methylene-, (1S)-	136.125	C_10_H_16_	1.96
30	2,6-Octadien-1-ol, 3,7-dimethyl-, acetate, (E)-	196.146	C_12_H_20_O_2_	2.82
31	Cyclohexane, 1-ethenyl-1-methyl-2,4-bis(1-methylethenyl)-, [1S-(1.alpha.,2.beta.,4.beta.)]-	204.188	C_15_H_24_	0.31
32	Caryophyllene	204.188	C_15_H_24_	1.53
33	Naphthalene, 1,2,3,5,6,7,8,8a-octahydro-1,8a-dimethyl-7-(1-methylethenyl)-, [1R-(1.alpha.,7.beta.,8a.alpha.)]-	204.188	C_15_H_24_	0.79
34	Naphthalene, 1,2,4a,5,8,8a-hexahydro-4,7-dimethyl-1-(1-methylethyl)-, (1.alpha.,4a.beta.,8a.alpha.)-(.+/-.)-	204.188	C_15_H_24_	0.63
35	2(3H)-Naphthalenone, 4,4a,5,6,7,8-hexahydro-4,4a-dimethyl-6-(1-methylethenyl)-, [4R-(4.alpha.,4a.alpha.,6.beta.)]-	218.167	C_15_H_22_O	2.82

## Data Availability

Some or all data, models, or code generated or used during the study are available from the corresponding author by request.
